# Construct Validity of the Athlete Introductory Movement Screen in Grassroots Footballers Aged 11–13 Years

**DOI:** 10.3390/children11070879

**Published:** 2024-07-19

**Authors:** Michael J Duncan, Matteo Crotti, Ricardo Martins, Lucas Guimaraes-Ferreira, Jason Tallis, William Pattison

**Affiliations:** 1 Centre for Physical Activity, Sport and Exercise Sciences, Coventry University, Coventry CV1 5FB, UK; matteo.crotti@unibg.it (M.C.); ae0282@coventry.ac.uk (R.M.); ad6463@coventry.ac.uk (L.G.-F.); ab0289@coventry.ac.uk (J.T.); pattisow@uni.coventry.ac.uk (W.P.); 2 Department of Human and Social Science, University of Bergamo, 24129 Bergamo, Italy

**Keywords:** motor skill, movement, pediatric, motor competence, validity

## Abstract

Background: This study examined the construct validity of the Athlete Introductory Movement Screen (AIMS) in children. Methods: Following ethics approval, parental consent, and child assent, 87 children (50 boys, 37 girls) aged 11–13 years (Mean ± SD = 12.4 ± 0.6 years) performed the AIMS and Test of Gross Motor Development (TGMD-3) in a counterbalanced order. AIMS tertiles were subsequently created, classifying children with ‘high’, ‘medium’, or ‘low’ movement skills. Results: A 2 (Gender) X 3 (AIMS tertile) ways analysis of covariance (ANCOVA), controlling for age and age at peak height velocity, with TGMD-3 scores as the dependant variable, indicated that TGMD-3 scores were significantly higher for girls categorised as having a medium movement skill compared to girls categorised as low, and those categorised having high movement skill compared to medium and low movement skill groups (all, *p* = 0.001). There was no difference in TGMD-3 scores for boys classed as having low and medium movement skills. Boys categorised as high for movement skills had significantly greater TGMD-3 scores than their peers categorised as having both low and medium movement skills (*p* = 0.001). Conclusions: As the AIMS differentiates the theoretically related construct of motor competence, this study demonstrates that the AIMS has construct validity as a measure of movement skill in children aged 11–13 years.

## 1. Introduction

The development of motor competence and effective movement skills during childhood are critical in enabling children to engage in lifelong physical activity and sports and particularly feature as key components of long-term athletic development models [1,2]. Lack of effective movement skills is one of the key barriers that prevent children from engaging in physical activity and sports [3], is associated with increased injury risk in sports [4], and is considered worldwide as ‘low’ or ‘poor’ for children and youth [5].

As a consequence, the assessment of movement skills in children and adolescents has become more common over the last decade [6]. Such movement screens are employed in various contexts with pediatric populations to assess athletic performance and injury risk in injury rehabilitation [7,8,9,10,11], in addition to being used in school Physical Education [3] and strength and conditioning to more effectively target training or pedagogical intervention [12,13]. The majority of movement screens are considered process-oriented assessments, assessing the quality of movement rather than the outcome (e.g., jump height, sprint speed). Such process-based assessments are important in the context of physical education and long-term athletic development as they provide contextual information related to movement deficits that may not be identified by product-oriented assessments.

Given the utility of movement assessment in youth populations, a variety of different movement screens have been employed in children and youth over the last twenty years. The most widely used and validated process-oriented assessment in the literature has been the Test of Gross Motor Development (second or third Editions [14]). The TGMD assesses gross motor competence comprising locomotor and object control skill subsets and is widely considered a measure of fundamental movement skills. Movement screens such as the Landing Error Scoring System [11] and Tuck Jump Assessment [10] have been validated to measure movement patterns associated with injury risk in pediatric populations. Furthermore, other authors have developed specific movement screens for golf [8] and netball [15] with youth participants for injury risk and talent identification. Likewise, there have been movement screens developed that assessed movement skills related to readiness for children to undertake resistance exercises, such as the Resistance Training Skills Battery for Children (RTSB; [16,17]), and movement skills related to potential athletic talent, such as the Athletic Ability Assessment [18]. For the most part, other than the TGMD, these aforementioned movement screens have tended to be used and developed with children already engaged in sports performance at a relatively high level, although construct validity of the RTSBc has been demonstrated in children who were ‘active’ but not necessarily engaged in formalised sports programmes at youth level [19].

More recently, the Athlete Introductory Movement Screen (AIMS, [20]) was developed as a means to address the shortfalls in previously established movement screens for children and youth by providing a brief but representative task specific to children and youth in the preliminary stages of or prior to a talent development pathway. The AIMS, comprised four individual movement skills, including locomotor and stability components, assessed key athletic movement skill competencies and was developed for use with junior athletes during adolescence with a view to athletes with appropriate movement skills undertaking introductory strength and conditioning [20]. The purpose of the AIMS, as proposed by the researcher who developed it [20], is to provide a common movement assessment that can be operationalised across athlete development settings. A secondary purpose of the AIMS is to increase the visibility and understanding of movement competencies in entry-level adolescents [20].

The AIMS was developed in a sample of 28 junior athletes (18 boys, 10 girls; mean age = 15.7 years), comprising four movements: overhead squat; push-up; lunge; front brace with shoulder touch [20]. Rogers et al. [20] concluded that the AIMS was a reliable and practical assessment tool for junior athletes. The results of their study demonstrated that the AIMS screen held good to high reliability as a sum score between and within raters and good reliability between multiple testing sessions. However, there remain key aspects of movement screen development that need to be considered before the AIMS can be more widely advocated for use in pediatric populations. A key next step is to determine the construct validity of the AIMS. No study to date has explored this issue, but for the AIMS to be considered a measure of movement skill in children, evidence of its validity is essential. Construct validity is particularly important in this context as it establishes whether the movement skill, as determined by the AIMS, differentiates theoretically related constructs that underpin athletic movement skills (e.g., motor competence) [21]. Moreover, it is important not to assume the utility of the AIMS based on a relatively small sample of adolescents who were also somewhat older than the age range, where long-term athlete development models suggested children should be starting to engage in formalised strength and conditioning training [1]. This is particularly so, given that one of the purposes of the AIMS screen is to determine readiness for children to engage in more formalised training, particularly strength and conditioning. The current study aimed to address this gap by examining the construct validity of the AIMS in children aged 11–13 years. Given that the AIMS is purported to assess movement competence, the present study hypothesised that children who scored highly on the AIMS would also score highly on an assessment of general motor competence.

## 2. Materials and Methods

### Participants

Eighty-seven children (50 boys, 37 girls) 11–13 years of age (Mean ± SD = 12.4 ± 0.6 years, 161.1 ± 8.8 cm, 49.9 ± 10.0 kg) who were regularly engaged (i.e., were registered and had played for a grassroots soccer team for at least one season) in grassroots soccer participated in this study. Children were recruited from grassroots clubs within Birming County FA. Institutional ethics approval, informed parental consent, and child assent were gained prior to data collection. Inclusion criteria comprised children who were registered for a grassroots soccer team (to comprise training and playing competitive games) within the English County FA structure and who had been registered within the English County FA structure for at least one year prior to participation. Children had to be between 11 and 13 years of age and free from injury at the time of participation. Any child who did not meet this criteria was excluded from participation. The mean ± SD of formalised playing experience was 4.2 ± 1.8 years, and the mean ± SD of time spent in grassroots soccer training and matches was 178.5 ± 38.6 min per week. The focus on grassroots soccer players in the current study was deliberate and directly aligned to the population the AIMS was purportedly developed for, i.e., children who are introductory athletes and not formally engaged in formalised athlete development activities. Likewise, the age of participants recruited in this study was deliberate, as the AIMS was designed for children in the stage where formalised strength and conditioning should be starting to take place within long-term athlete development models [1]. Thus, the choice to recruit children aged 11–13 years directly maps to this stage to examine the construct validity of the AIMS in a population of the age and developmental stage the measure was designed for. The Fédération Internationale de Football Association [22] defines grassroots soccer as recreational soccer taking place, predominantly in children from the age of 6 years on. The definition of grassroots soccer employed in the current study adhered to the FIFA definition [22].

## 3. Procedures

### 3.1. Experimental Design

Children attended the human performance laboratory, where they undertook anthropometric measurements, followed by performance of the AIMS and the TGMD-3. The performance of the AIMS and TGMD-3 was counterbalanced and separated by 30 min to ensure that ‘fatigue’ did not influence the performance of the two movement screens. Administration of both the AIMS and TGMD-3 followed recommended guidelines [14,20] for test administration, including familiarisation.

### 3.2. Anthropometry

Children were lightly dressed (shorts, t-shirt) and barefoot for anthropometric assessment, where height (cm), sitting height (cm), leg length (cm) and mass (kg) were assessed (to the nearest cm and 100 g) using a SECA stadiometer and weighing scales (SECA Instruments, Ltd., Hamburg, Germany). Age at peak height velocity (APHV) was determined using height, sitting height, leg length, body mass and chronological age as a measure of maturation using the Moore et al. prediction equation [23].

### 3.3. AIMS

The AIMS consists of four movement skills (overhead squat, push-up, lunge, and front brace with chest touches), with proficiency in these exercises indicating better athletic movement competence, providing the platform for engaging in more formalised movement training [20]. Procedures for familiarisation, administering, and scoring the AIMS were taken from the previously validated methodology for the AIMS (Rogers et al., 2019). The AIMS tasks were performed in a standardised order as follows and following procedures employed with the AIMS previously [20]: (1) overhead squats with a dowel rod; (2) full body push-ups; (3) lunges; and (4) straight-arm prone brace with alternating hand touches to the opposite shoulder (two per shoulder in a set of four repetitions).

Prior to performing each skill, the participants observed movement demonstrations and received verbal instruction related to the key points for each movement related to the scoring criteria [20]. After this and in line with prior work describing the administration of the AIMS, participants were permitted to practice four to five repetitions of the movements. Following familiarisation (1–2 min/movement), participants performed two sets of four repetitions of each movement. During administration and congruent with guidelines for administration for the AIMS [20], only questions relating to assessment protocol (e.g., number of repetitions) were answered during the assessment; encouragement but not skill-related feedback was given [17,24]. The first set was performed and video-recorded (Sony HDR-CX405, Sony, Weybridge, UK) in the frontal and sagittal plane. As per prior guidelines for administering the AIMS, participants were given 10 to 15 s between sets and 4 to 5 min between tasks. Subsequent scoring of the AIMS was performed retrospectively from the video footage via Quintic Biomechanics analysis software v21 (Quintic Consultancy Ltd., Coventry, UK). Raters were permitted to pause and rewatch the footage as many times as required to complete their scoring.

Scoring of the AIMS was completed in line with prior studies. Full details of the scoring protocol are presented in the paper by Rogers et al. [20]. Each of the skills had four subcriteria per task; each scored on a three-point scale: three points = movement position and/or control criterion achieved through each repetition in a set; two points = inconsistent form with only some correct repetitions or minor misalignments on all repetitions; one point = did not meet position and/or control criterion for any repetition. Scoring was out of 12 points per task, with a maximum of 48 points as a composite AIMS score. For the AIMS score, tertiles were created with children being classed as ‘high’, ‘medium’, or ‘low’ for athletic movement skills. Such a procedure has been employed by previous researchers [24,25]. The decision to use tertiles follows recommendations from the review data [26], suggesting that the analysis by skill tertiles provides an effective means to assess evidence for a proficiency barrier, which is particularly pertinent in establishing construct validity [27]. The creation of tertiles in this manner is not mandated by the guidelines for the administration of the AIMS [20]. However, creating tertiles representing high, medium and low AIMS performance enables the assessment of construct validity of the AIMS in a manner that is recommended by prior researchers [26] to establish the validity of motor skill assessments.

Two researchers who were experienced in assessing children’s movement skills scored the videos. These researchers were trained prior to scoring in two separate two-three hour sessions by watching videoed skills of children performing the AIMS and rating these against a previously determined ‘gold standard’. The process for AIMS scoring was congruent with procedures used for the assessment and scoring of motor competence and movement screens [19,28]. The training was considered complete when scores for the two trials from each observer differed by no more than one component per trial from the instructor’s score for each skill (>80% agreement). Inter- and intra-rater reliability analysis was performed on 10% of all the videos (i.e., 12 participants, across all skills, videos (randomly selected, every seventh participant). For intra-rater reliability, the coding of the videos was performed separately by the two researchers and then compared. Intraclass correlation coefficients for inter and intra-rater reliability were 0.914 (95% CI = 0.85–0.94) and 0.974 (95% CI = 0.93–0.98), respectively.

### 3.4. General Motor Competence

General motor competence was assessed using the Test of Gross Motor Development-3 (TGMD-2) [14]. In the current study, the following skills (three locomotors, three object controls) were assessed: run; jump; hop; catch; overhand throw; and underhand throw, on the basis that the PE curriculum in England for children in this age group focuses on children mastering these skills [29]. Each skill comprised three–four components and was video-recorded (Sony HDR-CX405, Sony, UK) and subsequently analysed using Quintic Biomechanics analysis software v21 (Quintic Consultancy Ltd., Coventry, UK). Scores from both skill attempts were summed to create a total (scored 0–46) overall raw score. Subtest scores for locomotor motor competence (0–24) and object control motor competence (0–22) were also created using the sum of the run, jump, and hop for locomotor motor competence, and the catch, overhand throw, and underhand throw for object control motor competence. In all cases, scores for total motor competence, locomotor, and object control motor competence followed the recommended guidelines for the administration and scoring of the TGMD-3 [14]. Analysis of movement skills for the TGMD-2 followed the same process described above for the AIMS. Inter- and intra-rater reliability analysis was performed for all the skills of the two researchers on 12 participants (A total of 14% of all the videos. Intraclass correlation coefficients for inter and intra-rater reliability were 0.925 (95% CI = 0.87–0.95) and 0.987 (95% CI = 0.94–0.98), respectively. The process followed was the same as that described for the reliability analysis of the AIMS.

### 3.5. Statistical Analysis

In order to examine whether there were any differences in general motor competence, locomotor competence, and object control competence as a function of AIMS scores, a series of analyses of covariance (ANCOVA) controlling for APHV was employed. Independent variables were motor competence scores, with dependent variables being gender and AIMS tertile. Given that the performance of general motor competence underpins the performance of more specific athletic movement skills assessed by the AIMS, by examining if TGMD-3 scores differed by AIMS tertile, construct validity could be assessed. Such a process has been used previously when establishing construct validity of other movement screens in children and youth [19,30]. Partial eta^2^ (Pη^2^) was used as a measure of effect size, and Bonferroni post hoc pairwise comparisons were used to examine where any differences lay. Data are presented as Mean ± SD. Statistical analysis was performed using SPSS Version 28 (Chicago, IL, USA). Statistical significance was set at a level of *p* < 0.05.

## 4. Results

**Figure 1:**
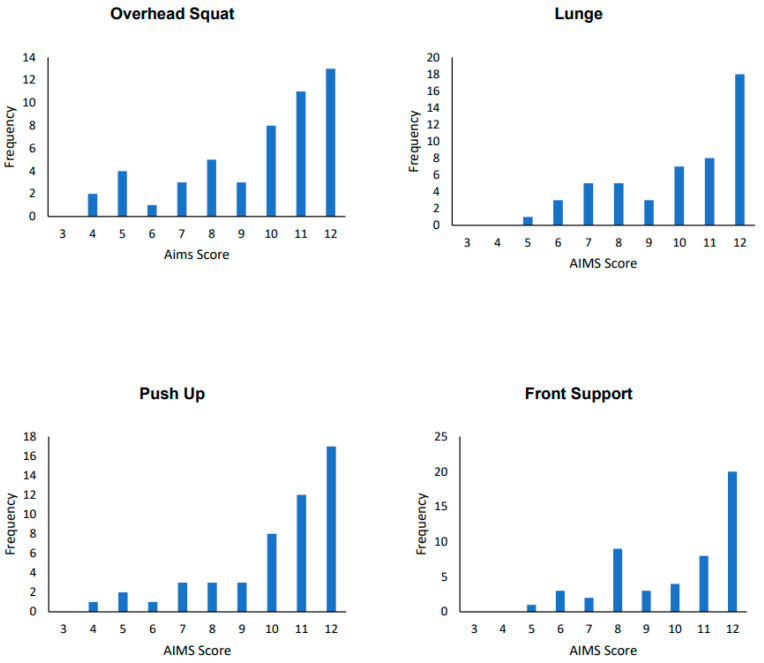
Score distribution from the 4-movement tasks within the AIMS. Each task is scored in four subcriteria and given 1, 2, or 3 points based on competency, with a possible total score range from 4 to 12.

The Mean SD and 95% Confidence Intervals for total FMS, locomotor FMS, and object Control FMS or boys and girls in Low, Medium, and High AIMS tertiles are presented in Table 1. Figure 1 shows the score distributions for movement tasks within the AIMS, with distributions for each of the four-movement tasks within the AIMS being positively skewed.

**Figure 2:**
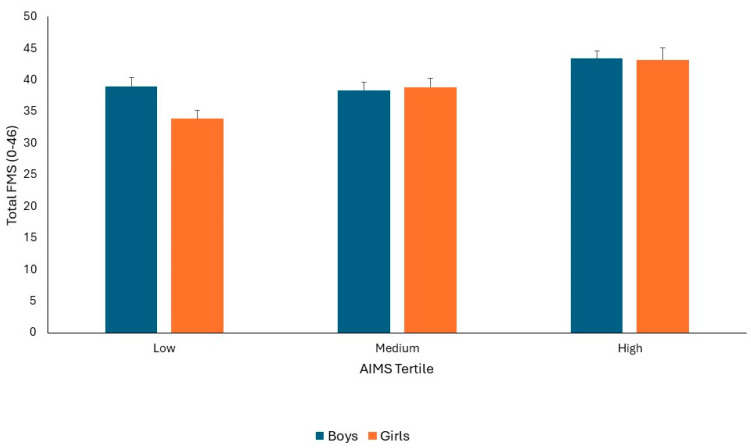
Mean ± SD of Total FMS scores split by sex and AIMS tertile groups.

For total FMS, ANCOVA controlling for maturation indicated a significant AIMS tertile by sex interaction (F1,82 = 3.372, *p* = 0.039, Pη^2^ = 0.078). Bonferroni post hoc pairwise comparisons indicated that boys had significantly better FMS compared to girls in low AIMS tertile (*p* = 0.001). There were no significant differences in FMS scores between boys and girls in the medium or high AIMS tertiles (Both *p* > 0.05). However, there were significantly better total FMS scores for girls in the medium and high AIMS tertiles, compared to the low (both *p* = 0.001) and between girls in the medium AIMS tertile, compared to girls in the high AIMS tertile (*p* = 0.001). For boys, there was no significant difference in total FMS score between the low and medium AIMS tertiles (*p* > 0.05), but boys in the high AIMS tertile had significantly better total FMS scores compared to boys in the medium and low AIMS tertiles (both *p* = 0.001). Maturation was not significant as a covariate (*p* > 0.05). The mean ± SD of total FMS scores split by sex and AIMS tertile groups is presented in Figure 2.

**Figure 3:**
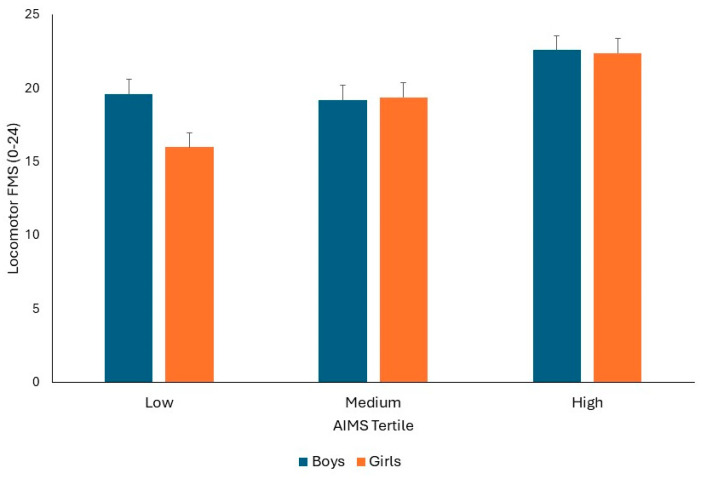
Mean ± SD of Locomotor FMS scores split by sex and AIMS tertile groups.

FMS data were subsequently split into locomotor and object control subsets and examined separately. For locomotor FMS, the covariate of maturation was not significant (*p* > 0.05), but there was a significant AIMS tertile by sex interaction (F1,82 = 3.196, *p* = 0.046, Pη^2^ = 0.074). Similar to the pattern observed for total FMS, Bonferroni post hoc multiple comparisons indicated that boys had significantly better locomotor FMS compared to girls in low AIMS tertile (*p* = 0.001). There were no significant differences in locomotor FMS scores between boys and girls in the medium or high AIMS tertiles (Both *p* > 0.05). There were significantly better locomotor FMS scores for girls in the medium and high AIMS tertiles, compared to the low (Both *p* < 0.001) and between girls in the medium AIMS tertile, compared to girls in the high AIMS tertile (*p* < 0.001). For boys, there was no significant difference in locomotor FMS score between the low and medium AIMS tertiles (*p* > 0.05), but boys in the high AIMS tertile had significantly better locomotor FMS scores compared to boys in the medium and low AIMS tertiles (both *p* < 0.001). The mean ± SD of locomotor FMS scores split by sex and AIMS tertile groups is presented in Figure 3.

**Figure 4:**
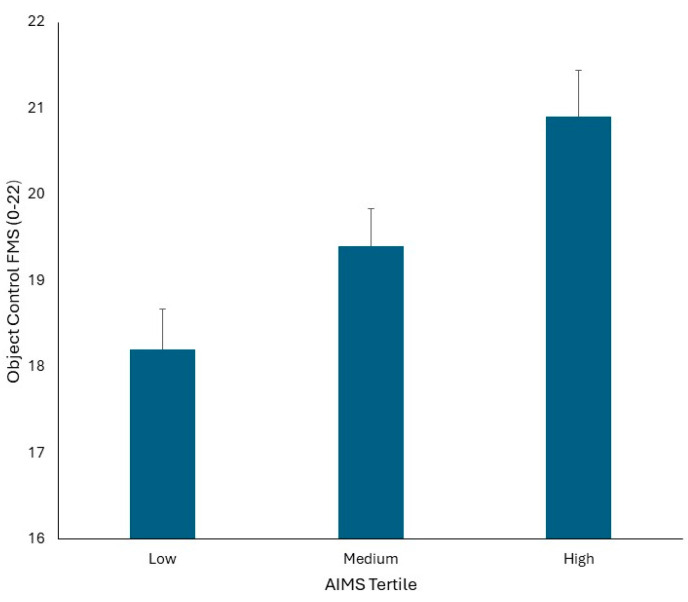
Mean ± SD of Object Control FMS scores split by sex and AIMS tertile groups.

When object control FMS scores were analysed, there was no significant effect of maturation as a covariate (*p* > 0.05). There was no significant AIMS tertile by sex interaction (*p* > 0.05) and no significant main effect for sex (*p* > 0.05). There was, however, a significant main effect for AIMS tertile (F1,80 = 6.829, *p* = 0.002, Pη^2^ = 0.146). Bonferroni post hoc pairwise comparisons indicated that irrespective of sex, children in the high AIMS tertile had significantly better object control FMS scores compared to those in the medium and low tertile groups (*p* = 0.01), and children in the medium AIMS tertile had significantly better object control FMS scores compared to those in the low AIMS tertile (*p* = 0.01). The mean ± SD of locomotor FMS scores split by sex and AIMS tertile groups is presented in Figure 4.

## 5. Discussion

The present study reports on the construct validity of the Athlete Introductory Movement Screen (AIMS) in children for the first time. The AIMS was developed as a practical assessment tool for emerging junior athletes [20], yet to date, no study has examined whether the AIMS demonstrated any form of validity, without which the researchers and practitioners cannot be confident that the tool assesses what it is purported to. By presenting this information, the present study addresses a key gap in relation to the psychometric properties of the AIMS as a movement screen for children and youth. The results of the present study suggest that scores on the AIMS differentiate total FMS, locomotor FMS, and object control FMS in children aged 11–13 years, and, therefore, by comparing AIMS with theoretically related constructs (i.e., FMS) that underpin athletic movement skills construct validity can be asserted [21].

The observation that children who were classed in the high AIMS tertile scored higher for total FMS, locomotor FMS and object control FMS compared to those in the medium and low AIMS tertile groups is reassuring in relation to the properties of the AIMS as a movement screen. This is because both the AIMS and TGMD are process-oriented and assess movement competency; so, for the AIMS to be valid, it should be expected that children who score higher on one screen will also score higher on the other assessment. The results of the current study suggest that for children aged 11–13 years, the AIMS shares some similarity with FMS in terms of the skills it seeks to assess, albeit that the AIMS and TGMD include different individual movements within their assessment. Of note, for total FMS and locomotor FMS scores, there were interactions between AIMS tertile and sex. For both total FMS and locomotor FMS, there were no differences in total FMS or locomotor FMS for boys and girls in the medium and high tertiles, but girls in the low FMS tertile had significantly poorer total FMS and locomotor FMS.

The original paper introducing the AIMS as a movement screen [20] presented data relating to the rater reliability of the AIMS as a movement assessment in a relatively small sample (*n* = 28) of children with a mean age of 15.7 years. Given the focus on the AIMS as a screen specifically for emerging junior athletes, the age of children assessed in work by Rogers et al. [20] is perhaps surprising. In the present study, children were specifically recruited within the ages where long-term athlete development models suggest that children should be starting to engage in formalised strength and conditioning training [1], the age range which the AIMS is purported to target. Moreover, unlike prior work, the analysis undertaken controlled for maturation, which is an important consideration given the age range and developmental stage that the AIMS was designed for. In the present study and the prior work by Rogers et al. [20], the distribution of AIMS scores across the four tasks within the screen was also examined. In the present study, the distribution of scores across all four tasks was positively skewed. This was not unexpected, as Rogers et al. [20] also demonstrated a positive skewing of score distribution in their study, and given the population recruited in the current study and also that of Rogers et al. (2019) were participating in youth sports, it might be assumed that those children would demonstrate better movement than a similar age population who did not participate in youth sports. The sample of youth who participated in the present study were, however, only involved in grassroots sport, i.e., they were not in any talent programme or professional youth academy as the current study sought to target our study to the demographic the AIMS was initially developed for. As a subsequent investigation from the present work, it would be interesting to examine AIMS performance in children not currently involved in youth sports. It is important to note that in the present study, we created tertiles reflecting low, medium, and high AIMS scores. Although this process creates three equal groups, reflecting low, medium, and high competence, it is specific to the population being examined.

## 6. Conclusions

The results of the present study suggest that the AIMS demonstrates construct validity as a measure of movement skill in children aged 11–13 years. By comparing scores on the AIMS to scores on the TGMD-3, a measure of general motor competence, evidence that the AIMS differentiates a theoretically related construct demonstrates the validity of the construct the AIMS purports to assess. Importantly, the present study demonstrates, for the first time, the validity of the AIMS in the age of the population the AIMS was specifically designed for. Coaches, physical education teachers, and practitioners working to improve movement skills in children might, therefore, consider the use of the AIMS for this purpose.

## Figures and Tables

**Table 1:** Mean SD and 95% Confidence Intervals for Total FMS, Locomotor FMS, and Object Control FMS or boys and girls in Low, Medium, and High AIMS tertiles.

								ANCOVA *		
		Low Aims Tertile		Medium AIMS Tertile		High AIMS Tertile		Sex	AIMS Tertile	Sex X AIMS Tertile
		M (SD)	95% CI	M (SD)	95% CI	M (SD)	95% CI	*p*	*p*	*p*
Total FMS (0–46)	Boys	39.0 (1.4)	36.1–42.0	38.4 (1.2)	36.1–40.8	43.4 (1.2)	40.9–45.8	0.117	0.001	0.039
	Girls	33.1 (1.3)	30.4–35.5	38.9 (1.4)	36.1–41.7	43.2 (1.9)	39.3–47.1			
Locomotor FMS (0–24)	Boys	19.6 (0.9)	17.6–21.3	19.1 (0.7)	17.6–20.5	22.6 (0.7)	21.2–24.1	0.096	0.001	0.046
	Girls	16.0 (0.8)	14.4–17.5	19.4 (0.8)	17.7–21.1	22.4 (1.2)	19.8–24.6			
Object Control FMS (0–22)	Boys	19.4 (0.7)	18.1–20.8	19.4 (0.6)	18.2–20.5	20.8 (0.6)	19.6–21.9	0.243	0.002	0.089
	Girls	16.9 (0.6)	15.7–18.2	19.5 (0.7)	18.2–20.9	21.0 (1.0)	19.1–22.9			

* Controlling for maturation.

## Data Availability

Data for this study are available from the principal authors upon reasonable request.
